# A mixed vertebrate eggshell assemblage from the Transylvanian Late Cretaceous

**DOI:** 10.1038/s41598-018-36305-3

**Published:** 2019-02-13

**Authors:** Mariela Soledad Fernández, Xia Wang, Mátyás Vremir, Chris Laurent, Darren Naish, Gary Kaiser, Gareth Dyke

**Affiliations:** 1 School of Biological Science and Technology, University of Jinan, Jinan, 250022 China; 2 Instituto de Investigaciones en Biodiversidad y Medioambiente, (INIBIOMA–CONICET), Centro Regional Universitario Bariloche (CRUB-UNCOMA), Quintral 1250, CP. 8400, San Carlos de Bariloche, Río Negro, Argentina; 3 Department of Natural Sciences, Transylvanian Museum Society (EME), 2-4 Napoca Street, Cluj-Napoca, 400009 Romania; 4 University of Southampton, SO17 1BJ Southampton, UK; 5 Department of Geology, Babeş-Bolyai University, RO-, 400006 Cluj Napoca, Kogălniceanu Street 1, Romania; 6 Royal British Columbia Museum, Victoria, BC Canada; 7 Evolutionary Zoology, University of Debrecen, Debrecen, Hungary

## Abstract

A Late Cretaceous-aged multi-taxon nesting site from Romania preserved in three dimensions reveals the earliest example of nest site sharing yet known from the vertebrate fossil record. Eggshell and osteological evidence combined in this single accumulation demonstrate that at least four vertebrate taxa including enantiornithine birds and another avian of indeterminate affinities as well as crocodylomorphs and gekkotan squamates nested together in the same place. Colonial nesting in enantiornithines was previously described from this site; here, we present the first fossil evidence that other vertebrates also nested in the same place, perhaps exploiting the presence of the large bird colony. We describe four distinct eggshell morphotypes that have been collected from this site and draw palaeoecological inferences based on this unique multi-taxon nesting association.

## Introduction

The eggs, hatchlings, nests, and nesting sites of extinct animals are relatively common in the vertebrate fossil record. Evidence of dinosaur reproductive activities have, for example, been interpreted, or inferred, from deposits that range in age from the Triassic to the Late Cretaceous, and are known from numerous sites around the world (*e*.*g*. Mongolia, China, Argentina, Montana, Portugal, and Romania)^1,2^. Although it remains debated exactly when ‘bird-like’ parental care evolved within archosaurs^3–6^, current evidence shows that it was present within non-avialan maniraptoran theropods (*e*.*g*. members of Alvarezsauridae, Oviraptorosauria, Dromaeosauridae, and Troodontidae)^7–9^ by the latest Cretaceous, 70 million years ago (Mya). Adult oviraptorosaurs are preserved in physical contact with neatly arranged eggs^10^, forelimbs protectively surrounding the clutch^11^, while fossil data shows that some non-avialan maniraptorans and Cretaceous stem-birds embedded their eggs individually into the substrate^9,10,12,13^ and produced precocial (fully-independent) young^14,15^.

Much less information is presently available for other non-avialan theropod dinosaur lineages although evidence is consistent with their use of reproductive modes similar to those of living turtles, crocodiles, or lacertans. However, with just a few exceptions (*e*.*g*. *Maiasaura*)^16,17^, evidence of significant hatchling parental care in dinosaurs is limited, although some groups appear to have nested in large colonies^13,18–20^. As far as it is known, both non-avialan theropod dinosaurs and Mesozoic birds had precocial young^2,10,13–15,21–27^; this contrasts with the altricial^3,28–31^ young of many living Aves where energetically demanding parental care is required. These behaviours include, but are not limited to, egg brooding and egg turning, both critical to improve hatching rate^32^, hatchling feeding, and the construction of complex nests^10,33^. Most significantly for palaeontological studies, the identifiable bones of precocial neonates have frequently been found in association with fossil eggs while altricial young do not develop hardened bones until after they hatch^3,33^. Their soft bones rarely form fossils.

The vertebrate record is also dominated by single-species egg and nest associations^1,2^, with a few rare exceptions of *in-situ* fossil eggs and eggshells (*e*.*g*.^34,35^). Among extant faunas, however, there are numerous examples of disparate taxa that share nesting areas and even the same nests; the South American gecko *Homonota darwinii*, for example, often lays eggs alongside nesting cormorants (*Phalacrocorax* spp.) and gulls (*Larus* spp.). These geckos utilise nesting areas for shelter, warmth, and the opportunity to feed on algae, which the birds use to construct their nests^36^. Similarly, the Lesser Rhea (*Pterocnemia pennata*) and Elegant Crested Tinamou (*Eudromia elegans*) often nest and brood alongside penguins in Patagonia with complete interspecific tolerance^37^. It is therefore parsimonious to predict that similar behaviours were also present in the past.

**Figure 1:**
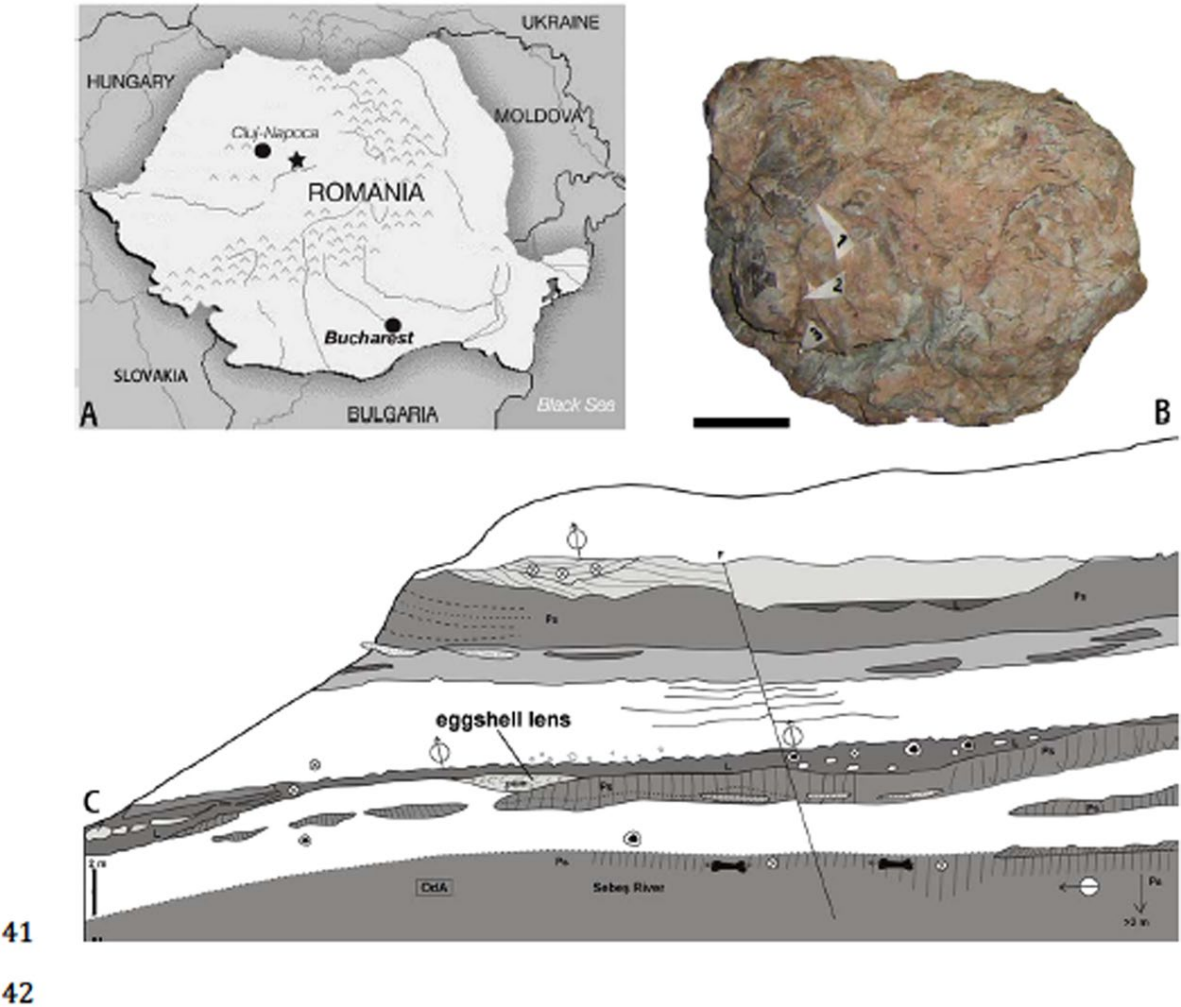
The site and accumulation. (**A**) Map showing the Late Cretaceous Od location (star), Sebes, Romania^26^. (**B**) One part of the Od calcareous lens as collected and prior to preparation. Scale bar is 5 cm. (**C**) Stratigraphic profile of Od/A outcrop in the Maastrichtian, Sebes Formation^38^.

Here, we discuss a fossil accumulation (Transylvanian Museum Society, Cluj Napoca, EME V.314) from the Late Cretaceous Oarda de Jos locality (Od) in the vicinity of the city of Sebeș, Transylvania (western Romania). This deposit contains at least four different eggshell morphotypes as well as complete eggs and isolated bones within a single, very restricted, micro-horizon (Fig. 1). In our initial reports^26,38^, we noted that this assemblage comprises a lens of calcareous mudstone that contains thousands of avian eggshell fragments and complete eggs. However, while the morphologies of adult and neonate bones found among the shell fragments are consistent with our earlier report of enantiornithine birds^26^, further preparation has revealed skeletal elements that cannot be assigned to that lineage, while including eggshell fragments from at least three additional vertebrate taxa. These include a bird of uncertain affinities, crocodylomorphs, and gekkotan squamates. Sedimentological and taphonomic evidence^35^ supports the conclusion that these additional vertebrate taxa were nesting within the same areas as the breeding enantiornithine colony, which makes the Od assemblage unique in the vertebrate fossil record.

## Results

### Taphonomy of the Od lens

As discussed in our initial reports^26,38^, the EME V.314 lens comprises a calcareous mudstone that contains thousands of eggshell fragments and a number of complete or near-complete eggs (N = 13) (Fig. 1). Observations show that the more complete eggs were deposited in the top third of the eggshell coquina, and that shell density within the lens approaches 80% of matrix volume (Fig. 1).

Sample E.V. 314 /1 includes 77 eggshells comprising about 60% (46 eggshells) horizontal fragments preserved concave side-up (CU), alongside 40% (N = 31) that are preserved concave side-down (CD). These subsets provide two distinct data samples for further statistical analysis; we therefore performed a proportion test on two samples considered representative of the whole (ratio: 60:40; X^2^ = 2.5455, 1 degree of freedom (d.f.), p = 0.59, 95% percent confidence interval (CI), between 0.4793 and 0.7056) that reveals that this distribution of shell orientations is similar to ratios seen at *in situ* avian nesting sites (ratio: 60:40; χ^2^ = 3.9781, 1 d.f.; p = 0.0461)^39,40^. Thus, for this phase of the burial event, it is clear the eggshell distribution included near complete, partially-crushed eggs preserved with their long axes orientated non-randomly.

Our observations also reveal the presence of a second phase event that accounts for 85 eggshells, of which 42% (N = 36) are preserved CU and 58% are preserved CD. A second proportion test (ratio: 42:58, χ^2^ = 1.6941, 1 d.f., p = 0.4235, 95% CI between 0.3185 and 0.5354) shows that this distribution of preserved shell orientations is similar to the eggshell ratios recorded at chick-trampled sites (ratio: 42:58; χ^2^ = 0.29, 1 d.f., p = 0.58; not significantly different; χ^2^ = 1.49, 1 d.f., p = 0.22). This result suggests that Od site shell orientations are consistent with interpretation as a chick-trampled, non-transported accumulation^40,41^. It is also noteworthy that the shell ratio at the Od site is significantly different from previously published fragment orientations, including samples buried within substrates (ratio: 38:62; χ^2^ = 0.02, 1 d.f., p = 0.86) and those that have been transported under experimental and natural fluvial conditions (ratio: 18:85; χ^2^ = 5.66, 1 d.f., p = 0.01)^39^.

A number of complete and identifiable adult enantiornithine bird bones are also mixed with smaller bones and numerous bone fragments within this lens (Fig. 1). These elements include two partial humeri, a complete coracoid, and an ulna which exhibits clear enantiornithine synapomorphies^26^. Half of the preserved long bones are inclined horizontally.

Our use of thin-sections and scanning electron microscope (SEM) images of samples from the EME V.314 lens augments our initial reports and reveals the presence of four distinct eggshell structural types (based on previously published terminologies and definitions)^42,43^: (1) Enantiornithine eggs; (2) Unidentified Ornithoid eggshell; (3) Krokolithid eggshell, and; (4) Gekkolithid eggshell.

### Eggshell descriptions

#### Enantiornithine eggs

**Figure 2:**
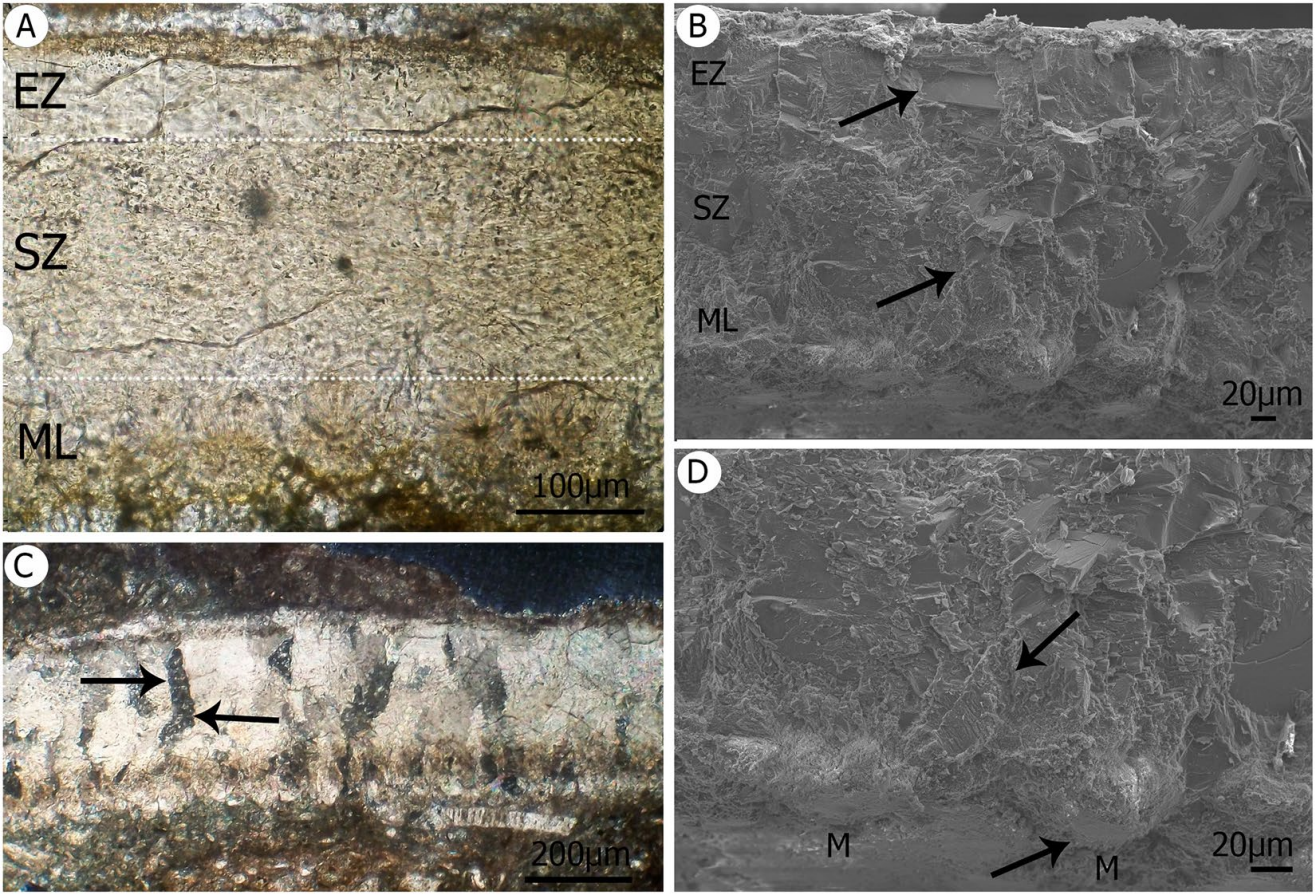
Enanthiornithine eggshell. (**A**) Thin section under PPL showing the thickness of the eggshell at low magnification. ML with barrel-shaped M, with its organic core at the base of the M. The CL overlies the ML and encompasses a SqZ and an EZ. The EZ consists of compact calcite crystals. (**B**) Eggshell under SEM, the lower arrow points the limit between two Ms. This image shows barrel-shaped mammilla. The transition between ML and the CL at low magnification is not evident, whereas the SqZ has squamatic ultrastructure it shows abrupt transition to the EZ. The upper arrow points the limit between two vertical crystals in the EZ. (**C**) Thin section under crossed Nicol prisms, arrow shows the limit of a prism from the CL (the shadow denotes a prism with irregular boundaries). (**D**) SEM shows details of the M, while the upper arrow points the arrangement of the wedges with tabular ultrastructure (See Fig. 3) with calcitic crystals diverge from the M base. The lower arrow indicates the M base including the replace organic core.

Description: Eggshells of this taxon are the most common. They comprise ca. 70% of all shells surveyed (N = 161 from 230 eggshells examined) and are all between 220 µm and 340 µm in thickness (Fig. 2A). It was originally reported that these shell lack ornamentation^26^, however subsequent examination of a larger sample suggests that the shell from the original sample has abraded external surfaces and in fact many of these shells actually exhibit a soft woven patina-like texture. One of the almost complete elongated eggs is 4.0 cm × 2.5 cm and would have had a volume of around 11 cm^3^. These eggs are slightly asymmetrical with one pole that is more pointed than the other.

**Figure 3:**
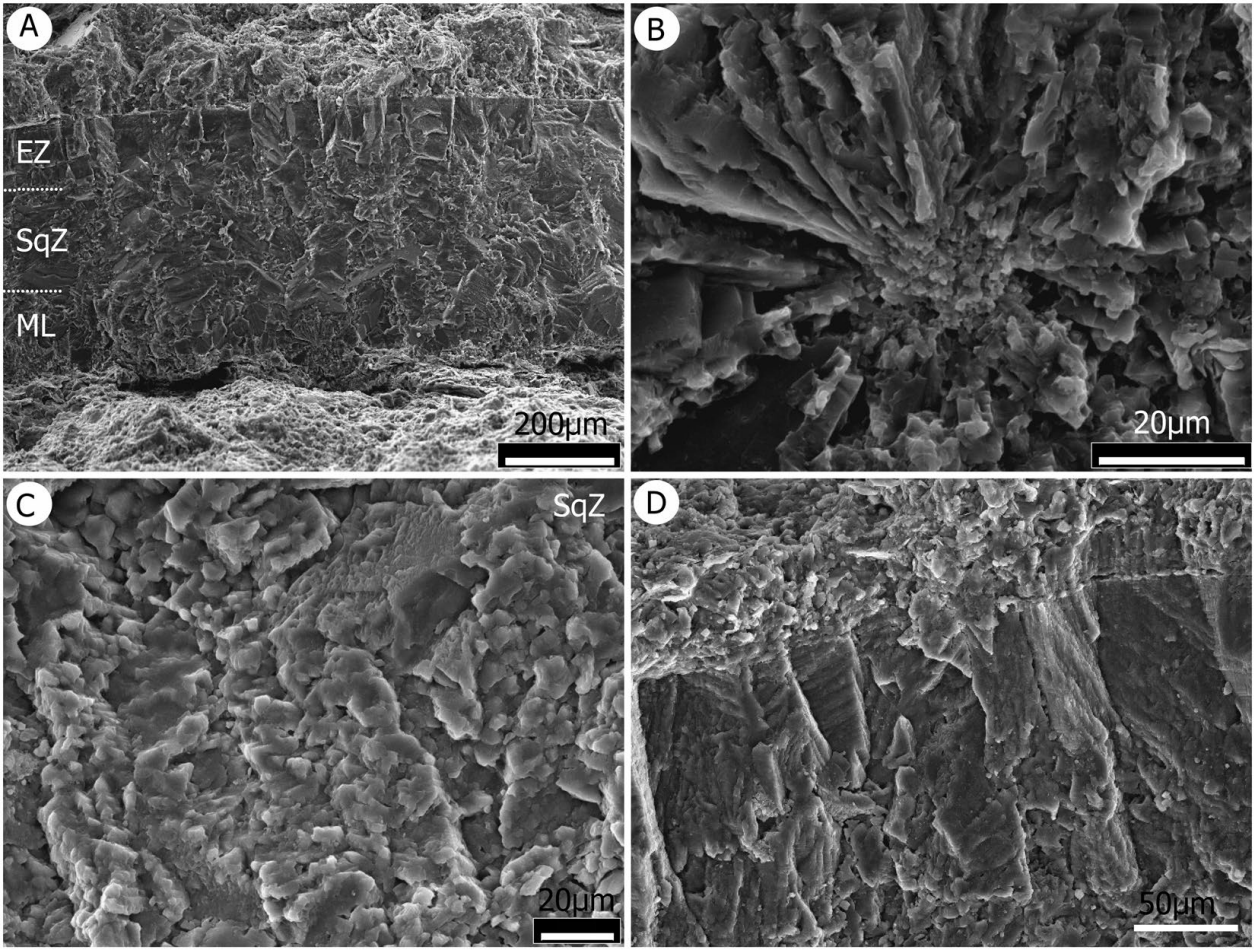
Enathiornithine eggshells. (**A**) Showing the thickness of the eggshell under SEM, at low magnification, and including an ML that has a barrel-shaped M with its organic core at the base of the ML. The CL overlies the ML in this case and encompasses a SqZ and EZ. External surface with a soft patination. (**B**) Details of the M base. (**C**) SqZ squamatic texture. (**D**) Vertical prisms within the EZ.

Our observations reveal that the microstructure of this shell type comprises two layers that encompasses a mammillary layer (ML) and a continuous layer (CL), a typical ornithoid basic form (Figs 2A and 3A). Mammillae are closely-packed, broad, barrel-shaped structures that are composed of calcite spherulites that extend outward from their base. These mammillae measure 75 μm to more than 80 μm in diameter (Fig. 2A,B).

Three general ultrastructural strata can be distinguished under SEM (Figs 2B and 3A); an organic core replaced by calcium (Fig. 2B), the base of this zone with radiating crystallites (Figs 2B and 3B), and examples of wedges with tabular ultrastructure (Fig. 2B). The CL comprises a squamatic zone (SqZ) with squamatic ultrastructure (SqU) and an external zone (EZ) with a more compact appearance and evident prisms (Fig. 3C,D). The SqZ is ca. 175 μm thick and gradually changes such that its boundary is difficult to distinguish. (Figs 2A–D and 3A). There are a number of tiny holes SqZ present which range in size between 0.3 μm and 1.5 μm (Figs 2B and 3C). The EZ under SEM appears as a graded transition to more compact calcite crystals that are blocky in appearance, with a vertical striation at high magnification (Fig. 3D). No squamatic texture is evident and the EZ which tends to have a more homogenous crystalline structure and is around 70 μm thick (Fig. 2A,B). Vertical prisms, longer than they are thick, are subtended from the external surface and form the external zone (Figs 2A,B and 3D).

Discussion: The enantiornithine bird eggs within EME V.314 are similar in volume to those of enanthiornithines from Neuquén City^13^, Argentina, and have similar shell thicknesses. These two types of eggs are also similar in shape to one another as both are asymmetrical and have pointed poles. An extensive bibliographic study carried out as part of this research (MF) reveals that the mammillary and continuous layers of extant paleognath eggshell typically comprise almost the entire shell thickness, while external zones and cuticle, if present, are reduced^44^. This is not the case for neognathous eggshells, which typically exhibit a squamatic layer much thicker than the mammillary, and which have a correspondingly thin external layer^44^. In contrast, Neuquén City eggs from the Argentine Bajo de la Carpa Formation (Río Colorado Subgroup) have been described with a ML and a SqZ that are proportionally thicker than the EZ^15,40^, while another Mesozoic bird egg^45^ has been reported with a ML which is 92.9 μm thick, a SqZ which is 58.7 μm thick, and an EZ which is 14.4 μm thick. A further example of an egg from the Brazilian Valle do Río do Peixe Formation (Turonian-to-Maastrichtian) has a ML, a SqZ, and EZ that are all equal in thickness, which has been interpreted as unique to this specimen^46^. Moreover, several authors have described extant neognath eggs with graded transitions between structural zones, while those of paleognaths always exhibit an abrupt transition^44^. Eggs assigned to *Gobipteryx*, as well as the Bajo de la Carpa and Oarda examples, all share a graded contact between their ultrastructural zones^46^. Three structural layers, together with prismatic transitions (graded transition), have also been described from Mongolian Cretaceous bird eggs^45^, and this has been cited as evidence for closer affinities with modern avians as opposed to basal ornithothoracines^45^, while the known Bajo de la Carpa eggs have been associated with Ornithothoracines on the basis of embryonic evidence from this Neognathous morphotype (prismatic condition)^15,44,46^ (Table 1). The contact between the eggshell zones in the Bajo de la Carpa eggshells is graded (not abrupt), as in the Od eggs in agreement with *Schweitzer et al*.^15^. It is clear that the Od eggs exhibit the same structural contact between their eggshell zones and have the same shape and volume of their Argentine counterparts. These Romanian eggs can therefore be assigned to enantiornithines, a conclusion which is further supported by their shell characteristics: two-layered ornithoid-basic type eggshell with subdivision of the second layer (CL) into to ultrastructural zones (SqZ and EZ) and the presence of bird bones within the assemblage (Table 1).

**Table 1:** Comparison between Romanian and enanthiornithid eggshells from around the world.

	Layers	CL to ML thickness ratio	Ornamentation	Shell volume	Thickness	system	Authors
Enanthiornithid eggshells	3	4 to 1	Smooth	11 cm^3^	240 µm–340 µm		This work
Enanthiornithid eggs from Patagonia	4	3 to 1	Smooth	17 cm^3^	300 μm		Schweitzer *et al*.^15^
Brazilian ornithothoracine egg	3	2 to 1	Smooth	6.38 cm^3^	125.5 μm		Marsola *et al*.^46^
Gobioolithid eggs	2	2 to 1	Smooth		100 μm to 400 μm	Angusticanaliculate	Mickailov (1996)
Ornitholithidae	2		Undulating ornamentation		1.9 mmto 4 mm	Rimocanaliculate	Dughi & Sirugue (1962)

#### Unidentified ornithoid eggshell

**Figure 4:**
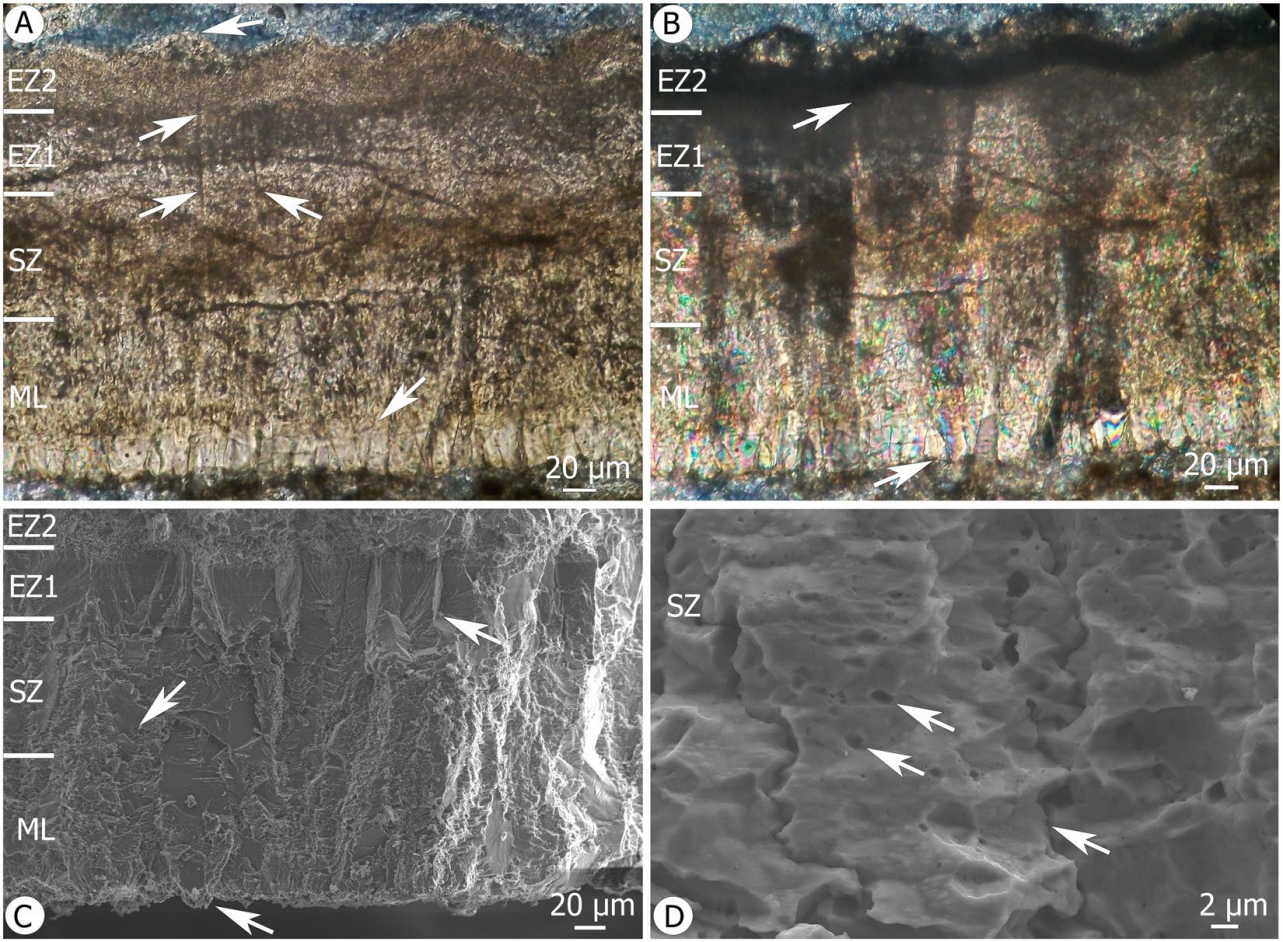
(**A**) Thin section of EME V.314 viewed under PPL reveals a four-layered eggshell. The ML is composed of slender M with a strong development. The lower arrow shows the transition between two M. CL is composed of a SqZ as well as EZ1 and EZ2. The CL has an irregular and very soft extinction pattern under crossed Nicols, and the upper arrows indicate the limit of two blocky crystals that comprise the part 1 of the EL. These crystals are longer than wide. The upper arrow points to the lower margin of the fourth layer, which looks similar to the SqZ but has an undulating external surface. (**B**) Under crossed Nicol prisms, a thin section reveals an irregular and very soft extinction pattern. Note that the fourth layer is completely opaque under PL. (**C**) SEM shows four layers: lower arrow points to a slender ML, the middle arrow indicates the SqZ, and the upper arrow points to the abrupt transition between the SqZ and part 1 of the EZ. Part 1 of the EZ in this case is composed of blocky calcite crystals, again longer than they are wide. The fourth layer is evident under SEM on top of the EZ part 1, with a similar ultrastructure of the SqZ. (**D**) SEM to show squamatic texture. The lower arrow denotes an opaque line which appears between each subunit of the squamatic ultrastructure, while the two upper arrows point to tiny holes which are distributed throughout the CL.

Description: Eggshells of this taxon are the least abundant within the Od accumulation, less than 1% of all shells surveyed. All of these specimens are between 240 µm and 260 µm in thickness (Fig. 4A) and have external surfaces that are ornamented with a rough covering of nodules. Thin sections of EME V.314 reveal that these eggshells are two-layered, and comprise a ML, a CL layer, which under SEM encompass two ultrastructural zones: a SqZ and an EZ external zone with the latter of which comprised of two parts, a ‘stony layer’^42^, and one with squamatic ultrastructure. The latter of these two is textured and forms the ornamentation; these shells possess a mammillary layer that is ca. 100 µm thick, and a CL of ca. 160 µm. The last one under SEM can be described as having: a SqZ that is ca. 100 µm thick, and an EZ with a ‘stony layer’ which is only 20 µm thick. The second part of this outer layer has a squamatic texture while undulating ornamentation occurs in the last ca. 40 µm (Fig. 4A).

The ML of these shells is comprised of slender mammillae measuring ca. 35 µm wide. Each has a width:height ratio of 0.35, which signifies strong and tall development. The second layer starts ca. 100 µm from the inner surface, and has a typically avian squamatic ultrastructure. An opaque line is present between each subunit on the SqZ (Fig. 4D). Tiny shell holes are preserved as spherical features unevenly distributed within the shell wall, particularly throughout the SqZ (Fig. 4D). The EZ of the third layer is comprised of blocky crystals 40 µm in width (Fig. 4C) that are longer than they are wide, and have a more compact crystal arrangement than the SqZ when viewed under SEM. These images also reveal that the transition between the mammillary layer and SqZ is not abrupt, but rather there is a soft transition between the two (Fig. 4C), while the second (SqZ) and third layers (EZ part 1) are abruptly delimited. SEM images reveal a fourth layer with squamatic texture, although this is opaque under plain polarised light (PPL) (Fig. 4B). It is noteworthy that this layer might also be the result of diagenesis; further work utilizing cathodoluminescence-approaches will be required to determine this (beyond the scope of this study).

Discussion: The ornithoid eggshells from the Od accumulation are not assigned in this study to any known oofamily, or oospecies. Nevertheless, we did compare them with shells from accipitriform neornithine birds^39^ because these are the only species that exhibit similar zonation; a ML followed by a CL with three different components, a SqZ, a ‘stony layer’^42^, and an external microcanaliculated zone^42^. The Od eggshells have different proportions between their eggshell layers, and a different ultrastructure in the fourth external zone. The ML in the Od eggshells comprises one third of the total thickness, while extant birds tend to have thinner ML. While extant birds have thinner, ca. 60 µm, ML, and the Romanian examples are 100 µm thick. Although the SqZ in the Od eggshells is similar in thickness to the ML, extant accipitriformes have a thin SqZ and also a third ‘stony layer’, which is unique among avian eggshells. Distinctly separated prisms have a homogeneous and solid ultrastructure. We have described the Od eggshells as having a more compact shape, but in these Romanian shells this layer is significantly thinner than in extant birds where it is 80 µm, and the fourth layer in neornithines has also been described as microcanaliculated. This reveals another important difference with the Od eggshells as they display a SqU and lack microcanaliculae; this region is smooth in extant birds but is undulated in the Od eggshells. As no fossil eggshells with these characters have been described, we consider that the Od shell of this type likely presents a unique combination of characters and therefore represent a new oospecies and oofamily. This will be addressed in our future work.

### Krokolithidae eggshells Kohring and Hirsch, 1996

#### Oospecies indet

**Figure 5:**
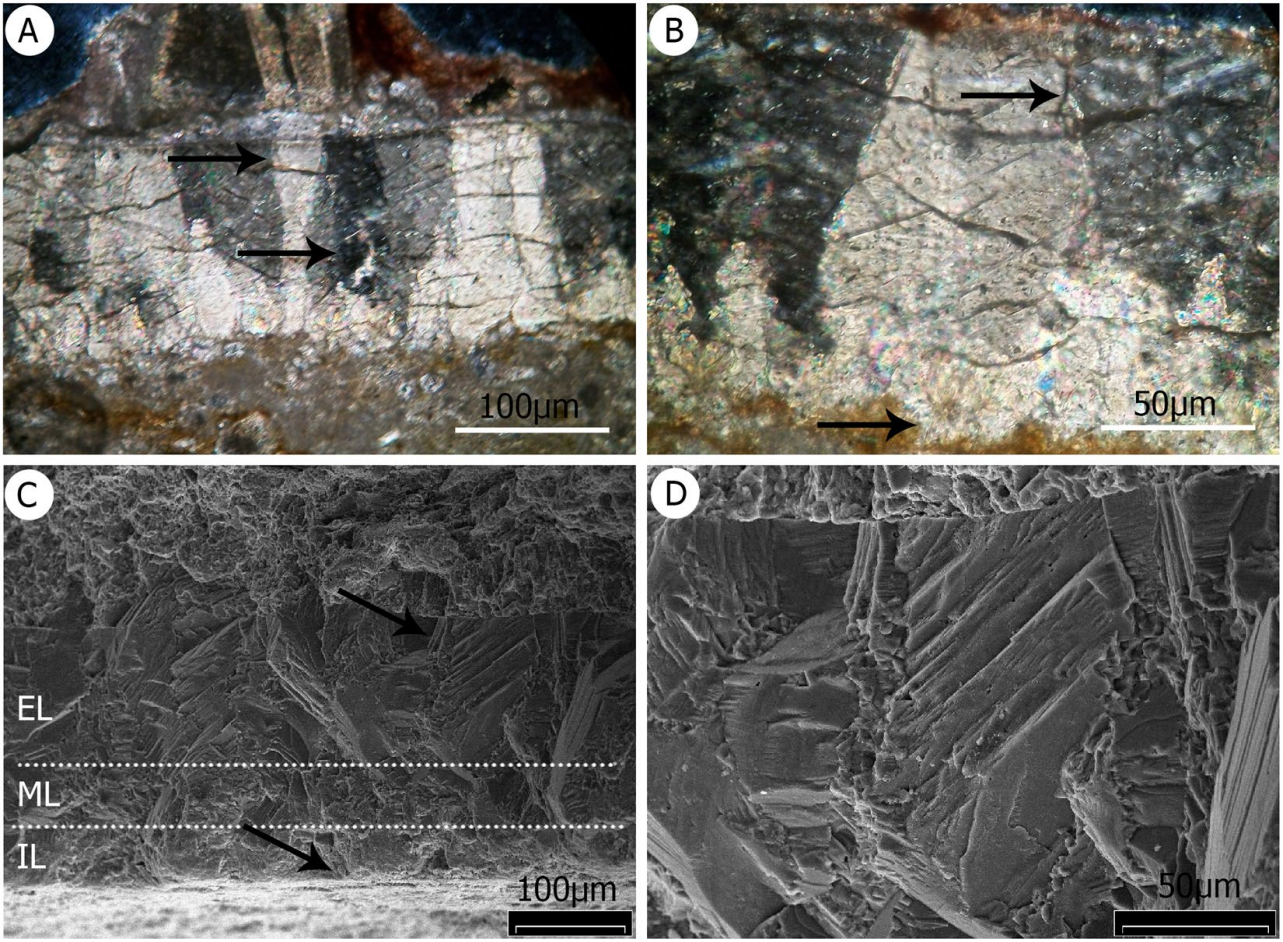
Crocodilian eggshell. (**A**) Thin sections show discrete, large trapezoidal-shaped shell units, composed of irregular wedges. Arrows show individual wedges visible in CPL. They exhibit the typical blocky extinction pattern. (**B**) Thin section under PPL, lower arrow shows that the BK contains a large aggregate of crystals and each has a bulbous base with a rosette-like structure. Upper arrow shows fish-bone pattern. (**C**) SEM showing three layers comprising the eggshell: IL, the inner layer, with the basal knob; ML, the middle layer that encompasses a thin honeycomb structure, and EL, external layer with calcite crystals with a more compact structure. Lower arrow indicates the basal knob, and upper arrow shows calcite cleavage plane. (**D**) SEM enlargement of EL (marked with an arrow); additional arrows pointing to cleavage planes of calcite crystals. All terminology follows previous work^19,42,54^.

Description: These shells are present in low numbers (*ca*. 28% of all shells surveyed, N = 45), have thicknesses between 250 µm and 275 µm, and comprise Crocodiliod basic shell type and Crocodiloid morphotype are used here as previously defined^42^. The discrete shell units in these specimens have a width:height ratio between 0.36 and 0.48 (Fig. 5A). Thin sections show discrete and approximately trapezoid units (Fig. 5A,B) that are composed of large and irregular wedges. Each shell unit is between 90 µm and 120 µm wide, with individual wedges between 50 µm and 75 µm (Fig. 5A,B) clearly visible in cross-polarised light. These units exhibit the typical blocky extinction pattern characteristic of crocodylomorph eggshells (Fig. 5A,B)^13,47–49^, with an inner layer that consists of a large aggregate of crystals (Fig. 5A,B). Each exhibits a bulbous base (Fig. 5B), where preserved, and SEM images reveal three distinct layers, a 48.63 µm thick inner layer, a middle 56.94 µm layer, and 144.83 µm thick outer layer with a densely calcified compact ultrastructure. Oblique lines present in this outer layer demark the cleavage planes of calcite crystals (Fig. 5C,D).

Discussion: This third shell morphology present in the EME V.314 lens is consistent with referral to crocodylomorph eggs^13,48–50^. The fossil record of Cretaceous crocodylomorph eggs and eggshells has been studied at a number of sites, including the Lower Cretaceous Glen Rose Formation^51^, the Upper Cretaceous Two Medicine Formation^19,52^, the Upper Cretaceous (Campanian) Fruitland Formation^53^, and the Upper Cretaceous Adamantina Formation, Brazil^54^. The Od eggshells, however, are distinct from the central Texan specimens which are between 600 µm and 700 µm thick^49^, while specimens from Montana^5^ are 655 μm thick and have smooth surfaces. The Od eggshells are thinner, between 150 µm and 275 µm think, but lack external ornamentation. Previous workers^5^ have also described how calcite spherulites in radial view on the inner eggshell surface extend outward from relatively evenly spaced nuclei approximately 390 μm apart. Although this pattern is also seen in the Od eggshells, it remains subtler; nuclei in this case are just 98 μm apart and the conical space between each basal knob is similar. Crocodile shells^50^ have also been described with two different ornamentations and different thicknesses, but are congruent with the Od specimens in ultrastructure and microstructure; the Romanian shells also lack external ornamentation, and have the same extinction pattern as the New Mexican eggshells. Thin sections of these also reveal horizontal accretionary lines, and both eggshells are thicker than shells at Od; Oliveira *et al*.^55^, described several near complete eggs that have external ornamentation not present in Od eggshells, and are thinner. Their thickness ranges between 150 µm and 250 µm. These materials reveal an undulating extinction pattern of irregular and divergently massive edges under PLM. Od eggshells do not have this undulating pattern. Under SEM, eggs from Adamantina Formation show radiating patterns of the crystalline wedges similar to Od eggshells. On the basis of shell thicknesses, the Od eggshells are similar to *Krokolites wilsoni*^49^ which has an eggshell thickness between 250 µm and 450 µm, *K*. *helleri*^48^ which is between 290 µm and 360 µm, and the Pai Mogo egg from the Jurassic^54^ which varies between 200 µm and 350 µm.

In earlier work, Ferguson^56^ described the structure and composition of eggshell membranes from the extant crocodylian *Alligator mississippiensis*. In living crocodylians, calcite crystals are deposited horizontally in eggshells, a pattern that is also seen in fossils from the Campanian-to-Maastrichtian transition of Spain^50^. Ferguson^56^ described an eggshell of five layers: an outer densely calcified layer (between 100 µm and 200 µm thick), a honeycomb layer (between 300 µm and 400 µm thick), an organic layer (between 8 µm and 12 µm thick), a ML (between 20 µm and 29 µm thick), and the eggshell membrane (between 150 µm and 250 µm thick). The Od material preserves three layers and organic membranes have not been preserved. It is not easy to identify all of these structural layers in fossil crocodylomorph eggshells because the organic material that forms layers in the live eggs is generally lost during fossilisation. Eggshell thickness of the Od crocodylomorph egg varies between 250 µm and 275 µm, and thus falls within the range of fossil crocodylomorph eggshells described by Hirsch and Kohring^56^. Most fossil crocodylomorph materials have been described as single layered eggshells, but extant eggs reveal a multilayered eggshell. These layers are not homologous with those observed in theropods (including birds). Notably, Moreno-Azanza *et al*.^50^ reviewed the terminology used to described the microstructure and ultrastructure of crocodylomorph eggshells and concluded that ‘all described crocodylomorph eggshell material, whether recent or fossil, displays at least two well-distinguished layers with distinct ultrastructure’. We observed triple-laminated eggshell under SEM (Fig. 5C), an inner layer, with or without a basal plate group which is ca. 50.2 µm thick, a middle layer with a thin honeycomb structure ca. 59.74 µm thick, and a more compact outer layer ca. 136.12 µm thick. The presence of an additional outer layer in crocodylomorph eggshells has not been noted in most literature, but is clearly present in some described ootaxa (e.g. *K*. *wilsoni*)^50^. The crocodylomorph eggshell from Od have three distinct layers and their blocky extinction pattern and basal knob support crocodylomorph affinities (Fig. 5C,D).

#### Gekkolithid eggshells

**Figure 6:**
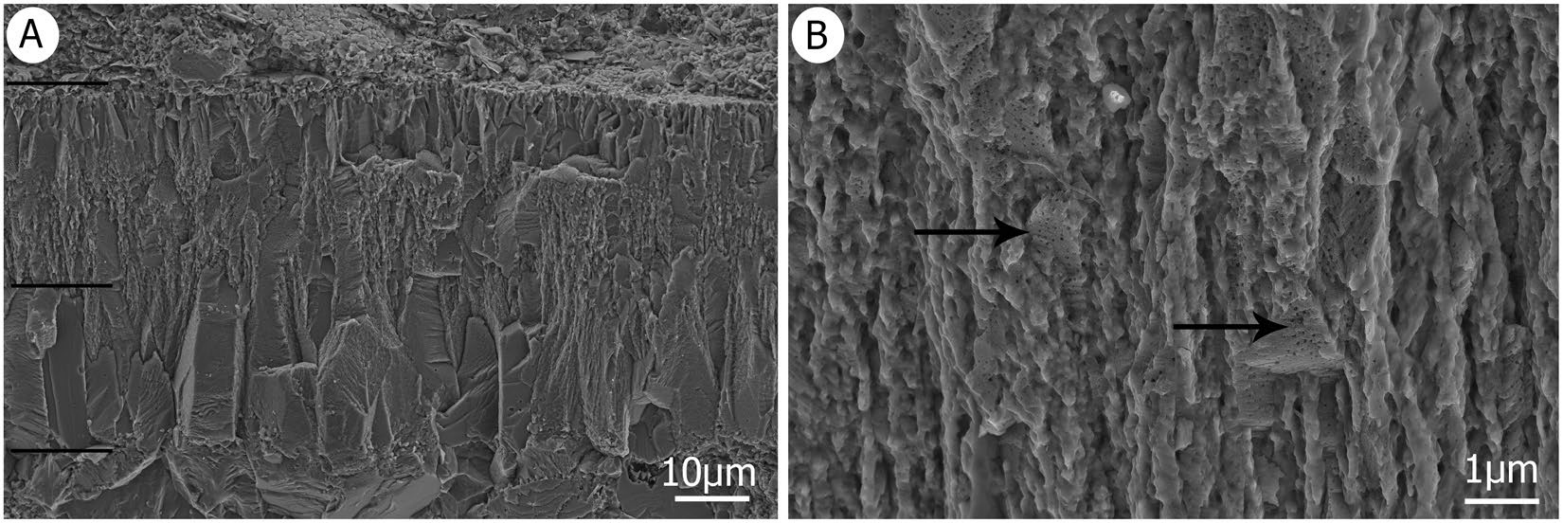
(**A**) SEM of a gekkotan eggshell with a two-layered eggshell structure. (**B**) SEM reveals the fine structure in the vertical prism of the second layer, arrows show abundant tiny holes, less than 0,01 microns in size.

Description: In total, about 1% of the Od shell fragments come are Geckoid basic type eggs^43^ and demonstrate the presence of gekkotans in the assemblage. Living gekkotans have small, thin-shelled eggs which are sub-spherical or ellipsoid in shape and have a smooth external surface^4^. In contrast, the eggshells from Od that have Geckoid basic eggshell type and Geckoid morphotype, are between 60 µm and 66 µm thick, and have two layers, the inner of which is wider than the outer, and both are characterised by numerous slender, densely-packed, and jagged columns (Fig. 6A). These columns lack cone-like morphologies, fan-like microstructure, and pore canals (Fig. 6A), but possess ultrastructural zonation (Fig. 6A). The inner layer comprises a compact, interlocking layer of calcite crystals with a prismatic appearance (not related to the prismatic layer of theropod eggshell) with lattices and etched faces (Fig. 6A,B). There is an abrupt transition between these crystals and acicular, parallel examples in the second layer that form a tightly packed palisade. The crystal ultrastructure is jagged, and is studded with abundant tiny holes (Fig. 6B) that resemble the primary vesicles (spherical films) seen in recent avian eggshells, although they are much smaller^48^. The organic core and basal plate group are also lacking in these shells (Fig. 6A).

Discussion: The geckoid-type shells from Od resemble those of the extant gecko *Ptydactylus*^57^, (Schleich and Kastle 1988 pp. 56, 57) as both have two distinct layers visible under SEM. Of these, the inner layer is comprised of blocky crystals which are longer than they are wide, while the external layer has thinner crystals with jagged boundaries, similar to those from Od^57^. Spanish fossil geckoid-type eggshells^58^ also have a particularly thin second layer, about 20 µm thick, much like the external layer seen in extant geckos^58^. Additional examples of these shells^58^ have been described as a single layer with a diagenetic second layer. They contrast with the Od eggshells which have two distinct layers. The remarkable structural similarities seen between the fossil shell and modern geckoid-type eggshell enable the clear assignment of these specimens to gekkotans, specifically Oofamily Gekkoolithidae Hirsch 1996.

## Discussion

Previous work has shown that eggshell orientation can be used to distinguish nesting and predation localities from transported assemblages in the fossil record^18,39–41,59^. Specifically, non-transported eggshell fragments at hatching and predation sites tend to rest CU rather than CD^39^. Therefore, trampled fragments and fragments transported by wind and water favour CD over CU orientations^40,60,61^. The CU:CD orientation ratio (42:58) in our study is statistically distinguishable from both the 60:40 ratio typical of *in situ* nests and the 15:85 ratio of transported nests when evaluated with a chi-square test^60,62^ and from those nests that are buried under substrate with a relation of CU:CD orientation (38:62). Our observed ratios (42:58) resemble the ratios (42:58) of chick-trampled eggshells, in accordance with the sedimentology and taphonomy of the Od lens which suggests that eggs were deposited in a single flood event. We have previously argued that these eggshells were transported a short distance and deposited in a shallow pond^26,61^. The results also support the conclusion that a proportion as high as 85% of CD eggshells in a fossil assemblage could be used to indicate transport regardless of eggshell type or substrate^61^, but a lower proportion should be used with caution. Again, as suggested by Hayward *et al*.^60^, palaeontologists should interpret the taphonomy of fossil eggshells with care and pay detailed attention to their sedimentological context^60^.

The EME V.314 Od accumulation contains eggshells consistent with four distinct vertebrate taxa, enantiornithine birds, birds of undetermined affinities, crocodylomorphs, and gekkotan lizards. Enantiornithine birds were the dominant taxon and nested at this site in large numbers. They may have actively accumulated the eggs or shells of other taxa, perhaps as a food source or a calcium bank. We consider this to be unlikely, however, as no gastric-acid etching (caused by digestion) was detected on the Od eggshells. It is more likely that these fossil eggshells, preserved alongside the complete bones and eggs of enantiornithines, evidence a mixed nesting association of at least four distinct taxa. This hypothesis is also consistent with sedimentological and taphonomic evidence^26^. The Od accumulation is thus unique in the vertebrate fossil record and represents the earliest record of disparate animals sharing the same nesting area.

We speculate that perhaps a plain area, created by seasonal flooding, offered the enantiornithines safety from predators and that their nest environments afforded shelter to smaller reptiles that benefitted from the security provided by the birds guarding their own nests^13^. This is often the case in extant mixed-nesting assemblages (see above). The enantiornithine component in the accumulation far outweighs the birds with undefined affinities. The presence of crocodylomorph and gekkotan material perhaps suggests that these animals were not only tolerated, but were perhaps not perceived as a threat to enantiornithine eggs or nestlings.

The sedimentology of the Od locality is consistent with a flood plain environment^26,38^ and there is no evidence of other nests. Indeed, while the only other known enantiornithine breeding associations are monospecific, resident birds nevertheless share these general areas with other vertebrates including non-avian theropods and crocodiles^13^.

## Materials and Methods

The lens containing the Od accumulation was collected in pieces by MV from the basal fluvio-paludal part of the Oarda outcrop (Maastrichtian Sebeș Formation) (Fig. 1C) and is housed in the Transylvanian Museum Society, Cluj-Napoca, Romania as EME V.314^26,38^.

All eggshell samples were treated with 10% acetic acid and the concretion of eggshell and matrix from the Od accumulation was then prepared as standard 30 μm petrographic thin-sections and examined with a polarised light microscope (Centro Atómico Bariloche, Argentina). Two other concretion samples were sputtered with 10 nm of platinum and analysed with an FEI (Hillsborough, Oregon) Nova NanoSEM 230 SEM at 15 kV. Images were evaluated using the open source FIJI software package^55^ to measure eggshell structural attributes^55^. We compared eggshell orientation ratios using a chi-square test, and orientations were measured from the horizontal face of the EME V.314/1 with an area of 11 × 15 cm. and EME V.314/2 with an area of 11 cm × 13 cm.

## References

[1] Carpenter, K. *Eggs*, *Nests*, *and Baby Dinosaurs* (Indiana University Press, 1999).

[2] Horner JR, De Ricqlès A, Padian K (2000). Long bone histology of the hadrosaurid dinosaur *Maiasaura peeblesorum*: growth dynamics and physiology based on an ontogenetic series of skeletal elements. J. Vertebr. Paleontol..

[3] Burley NT, Johnson K (2002). The evolution of avian parental care. Philos. Trans. R. Soc. Lond. B. Biol. Sci..

[4] Cockburn A (2006). Prevalence of different modes of parental care in birds. Proc. Biol. Sci..

[5] Jackson FD, Horner JR, Varricchio DJ (2010). A study of a *Troodon* egg containing embryonic remains using epifluorescence microscopy and other techniques. Cretac. Res..

[6] Wesolowski T (2004). The origin of parental care in birds: A reassessment. Behav. Ecol..

[7] Agnolin FL, Powell JE, Novas FE, Kundrát M (2012). New alvarezsaurid (Dinosauria, Theropoda) from the uppermost Cretaceous of north-western Patagonia with associated eggs. Cretac. Res..

[8] Norell MA, Clark JM, Chiappe LM, Dashzeveg D (1995). A nesting dinosaur. Nature.

[9] Varricchio DJ, Jackson FD, Trueman CN (1999). A nesting trace with eggs for the Cretaceous theropod dinosaur. Troodon formosus. J. Vertebr. Paleontol..

[10] Varricchio DJ, Jackson F, Borkowski JJ, Horner JR (1997). Nest and egg clutches of the dinosaur *Troodon formosus* and the evolution of avian reproductive traits. Nature.

[11] Norell MA, Clark JM, Chiappe LM (2001). An embryonic oviraptorid (Dinosauria: Theropoda) from the Upper Cretaceous of Mongolia. Am. Museum Novit..

[12] Sabath K (1991). Upper Cretaceous amniote eggs from the Gobi Desert. Acta Palaeontol. Pol..

[13] Fernández MS (2013). A large accumulation of avian eggs from the Late Cretaceous of Patagonia (Argentina) reveals a novel nesting strategy in Mesozoic birds. PLoS One.

[14] Elzanowski A (1981). Embryonic bird skeletons from the Late Cretaceous of Mongolia. Palaeontol. Pol..

[15] Schweitzer MH (2002). Late Cretaceous avian eggs with embryos from Argentina. J. Vertebr. Paleontol.

[16] Horner JR, Makela R (1979). Nest of juveniles provides evidence of family structure among dinosaurs. Nature.

[17] Varricchio DJ, Barta D (2015). Revisiting Sabath’s ‘Larger Avian Eggs’ from the Gobi Cretaceous. Acta Palaeontol. Pol..

[18] Hedricka BP (2014). The osteology and taphonomy of a *Psittacosaurus* bonebed assemblage of the Yixian Formation (Lower Cretaceous), Liaoning, China. Cretac. Res..

[19] Fastovsky DE (2011). A nest of *Protoceratops andrewsi* (Dinosauria, Ornithischia). J Paleontol..

[20] Varricchio DJ, Martin AJ, Katsura Y (2007). First trace and body fossil evidence of a burrowing, denning dinosaur. Proc R Soc B.

[21] Horner JR, Weishampel DB (1988). A comparative embryological study of two ornithischian dinosaurs. Nature.

[22] Hirsch KF, Quinn B (1990). Eggs and eggshell fragments from the Upper Cretaceous Two Medicine Formation ofMontana. J. Vertebr. Paleontol..

[23] Varricchio DJ, Horner JR, Jackson FD (2002). Embryos and eggs for the Cretaceous theropod dinosaur Troodon formosus. J. Vertebr. Paleontol..

[24] Zhou Z, Zhang F (2004). A precocial avian embryo from the Lower Cretaceous of China. Science.

[25] Buffetaut E (2005). Minute theropod eggs and embryo from the Lower Cretaceous of Thailand and the dinosaur-bird transition. Naturwissenschaften.

[26] Dyke G, Vremir M, Kaiser G, Naish D (2012). A drowned Mesozoic bird breeding colony from the Late Cretaceous of Transylvania. Naturwissenschaften.

[27] Varricchio DJ, Jackson FD (2004). A phylogenetic assessment of prismatic dinosaur eggs from the Cretaceous Two Medicine Formation ofMontana. J. Vertebr. Paleontol..

[28] Kucita P, Wang SC, Li WS, Cook RB, Starink MJ (2015). Microstructure characterization of hypereutectoid aluminium bronze composite coating. J. Phys. Conf. Ser..

[29] Starck, J. M. *Avian Growth and Development: Evolution within the Altricial-Precocial Spectrum* (Oxford University Press, 1998).

[30] Starck JM (1993). Evolution of avian ontogenies. Curr. Ornithol..

[31] Nice, M. M. *Development of Behavior in Precocial Birds* (Linnaean Society of New York, 1962).

[32] Deeming, D. C. In Egg incubation: its effects on embryonic development in birds and reptiles. (Deeming, D. C. and Ferguson, M. J. eds). 307–232 (Cambridge University Press, 1991).

[33] Dyke GJ, Kaiser GW (2010). Cracking a developmental constraint: egg size and bird evolution. In Proceedings of the VII International Meeting of the Society of Avian Paleontology and Evolution, (W. E. Boles & T. H. Worthy.ed) – *Au*st. *M*useum. Rec..

[34] Coria RA, Salgado L, Chiappe LM (2010). Multiple dinosaur egg-shell occurrence in an Upper Cretaceous nesting site from Patagonia. Ameghiniana.

[35] Fernández, M. S. & García, R. A. Parasitism in dinosaur clutches? VI International symposium about dinosaur palaeontology and their environmens. Salas de los Infantes, Burgos (2013).

[36] Scolaro, J. A. Personal communication (2015).

[37] Scolaro, J. A. Ecología de la nidificación del pinguino de Magallanes (*Spheniscus magellanicus*) en la colonia de Punta Lobería, Chubut, Argentina/. (Centro Nacional Patagónico, 1985).

[38] Vremir M (2010). New faunal elements from the Late Cretaceous (Maastrichtian) continental deposits of Sebeş area (Transylvania). Acta Musei Sabesiensis.

[39] Hayward JL (2000). Eggshell taphonomy at modern gull colonies and a dinosaur clutch site. Palaios.

[40] Wang PL, Jackson FD, Varricchio DJ (2013). Nest taphonomy of common terns (*Sterna hirundo*) on Poplar Island, Chesapeake Bay, Maryland. Hist. Biol..

[41] Jackson FD, Varricchio DJ, Jackson RA, Walde AD, Bishop GA (2015). Taphonomy of extant desert tortoise (*Gopherus agassizii*) and loggerhead sea turtle (*Caretta caretta*) nesting sites: Implications for interpreting the fossil record. Palaios.

[42] Mikhailov, K. E. *Avian Eggshells: An Atlas of Scanning Electron Micrographs* (British Ornithologists’ Club, 1997).

[43] Mikhailov, K. E. *Fossil and Recent Eggshell in Amniotic Vertebrates: Fine Structure*, *Comparative Morphology and Classification* (The Paleontological Association, Special Paper in Palaeontology No. 56., 1997).

[44] Grellet-Tinner G (2006). Phylogenetic interpretation of eggs and eggshells: Implications for phylogeny of Palaeognathae. Alcheringa An Australas. J. Palaeontol..

[45] Grellet-Tinner & Norell (2002). An avian egg from the Campanian of Bayn Dzak, Mongolia. J. Vertebr. Paleontol.

[46] Marsola *et al*. The first fossil avian egg from Brazil. Alcheringa: An Australasian Journal of Palaeontology, 10.1080/03115518.2014.926449 (2014)

[47] Hirsch, K. F. In *The**Palaeobiology of Trace Fossils* (Donovan, S. K. ed.) 269–294 (John Wiley and Sons, 1994).

[48] Kohring R, Hirsch KF (1996). Crocodilian and avian eggshells from the Middle Eocene of the Geiseltal, Eastern Germany. J. Vertebr. Paleontol..

[49] Hirsch KF (1985). Fossil crocodilian eggs from the Eocene of Colorado. J. Paleontol..

[50] Moreno-Azanza M, Bauluz B, Canudo JI, Puértolas-Pascual E, Sellés AG (2013). A re-evaluation of aff. Megaloolithidae eggshell fragments from the uppermost Cretaceous of the Pyrenees and implications for crocodylomorph eggshell structure. Hist. Biol..

[51] Rogers JV (2001). A complete crocodiloid egg from the Lower Cretaceous (Albian) Glen Rose Formation, Central Texas. J. Vertebr. Paleontol..

[52] Jackson FD, Varricchio DJ (2010). Fossil Eggs and Eggshell from the Lowermost Two Medicine Formation of Western Montana, Sevenmile Hill Locality. Journal of Vertebrate Paleontology.

[53] Tanaka K, Zelenitsky DK, Williamson T, Weil A, Therrien F (2011). Fossil eggshells from the Upper Cretaceous (Campanian) Fruitland Formation, New Mexico. Hist. Biol..

[54] Oliveira CEM (2011). Crocodylomorph eggs and eggshells from the Adamantina Formation (Bauru Group), Upper Cretaceous of Brazil. Palaeontology.

[55] Schindelin J (2012). Fiji: An open-source platform for biological-image analysis. Nature Methods.

[56] Hirsch KF, Kohring R (1992). Crocodilian eggs from the middle Eocene Bridger Formation, Wyoming. J. Vertebr. Paleontol..

[57] Schleich H. H., Kästle W. *Reptile Egg-Shells: SEM Atlas* (Gustav Fischer Verlag, 1988).

[58] Kohring R (1991). Lizard egg shells from the Lower Cretaceous of Cuenca Province, Spain. Palaeontology.

[59] Nys Y, Gautron J, Garcia-Ruiz JM, Hincke MT (2004). Avian eggshell mineralization: Biochemical and functional characterization of matrix proteins. Comptes Rendus Palevol..

[60] Hayward JL, Dickson KM, Gamble SR, Owen AW, Owen. KC (2011). Eggshell taphonomy: environmental effects on fragment orientation. Hist. Biol..

[61] Imai T, Varricchio DJ, Cahoon J, Plymesser K (2015). Sedimentological analyses of eggshell transport and deposition: Implication and application to eggshell taphonomy. Palaios.

[62] Ferguson MWJ (1981). The structure and composition of the eggshell and embryonic membranes of *Alligator mississippiensis*. Trans. Zool. Soc. Lond..

